# Supplementary material for: Excess S-adenosylmethionine inhibits methylation via catabolism to adenine
Source: Commun Biol. 2022 Apr 5;5:313. doi: 10.1038/s42003-022-03280-5 (PMC8983724; doi:10.1038/s42003-022-03280-5)
Supplement: Supplementary file 2 — Supplementary Information [file 42003_2022_3280_MOESM2_ESM.pdf]

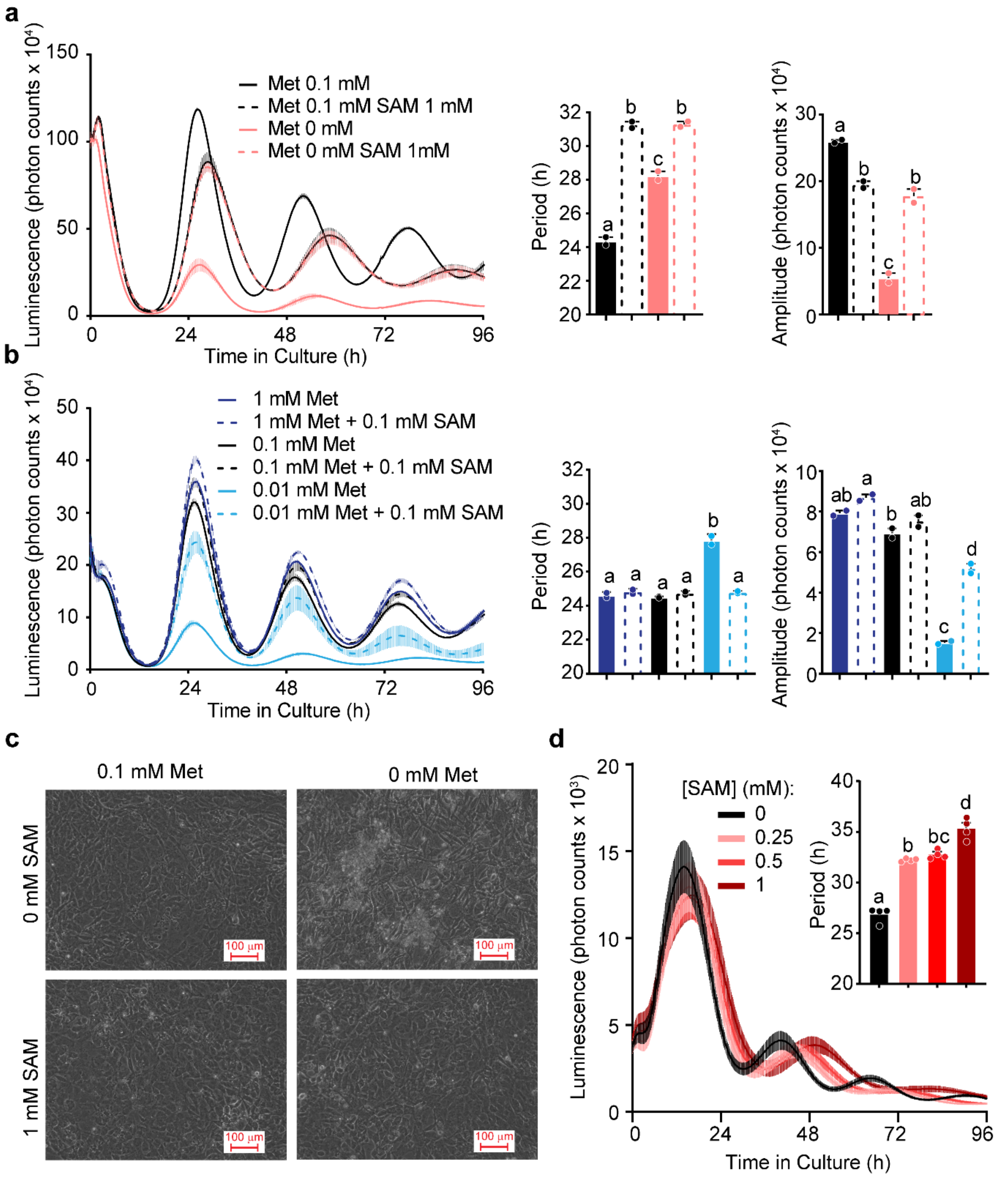

Supplementary Figure 1: Exogenous SAM partially rescue cells from the lack of methionine. **a**, 0.1 mM SAM rescues PER2::LUC mouse embryonic fibroblasts from the effects of 0.01 mM methionine on the period and amplitude of PER2::LUC oscillations. **b**, 1mM SAM overrides the effects of methionine deprivation on PER2::LUC mouse embryonic fibroblasts, clamping oscillations at a fixed period length and amplitude. **c**, 1mM SAM rescue cells from the lack of methionine. Notice the confluent cell population with 1 mM SAM/0 mM methionine but the less crowded cells without SAM or methionine, with clumps of dead cells. **d**, SAM dose-dependently lengthens the circadian period of human U-2 OS cells expressing bioluminescent reporter for the expression of the core clock gene *Bmal1* (aka *Arntl*). All bar graph data shown are mean +/- SEM of 2 (panel a, b) or 4 (panel d) culture wells. Data shown as bar graphs analysed by One-Way ANOVA followed by Bonferroni multiple comparison test, a vs. b vs. c vs. d (labels at the top of each bar) at least $p<0.01$.

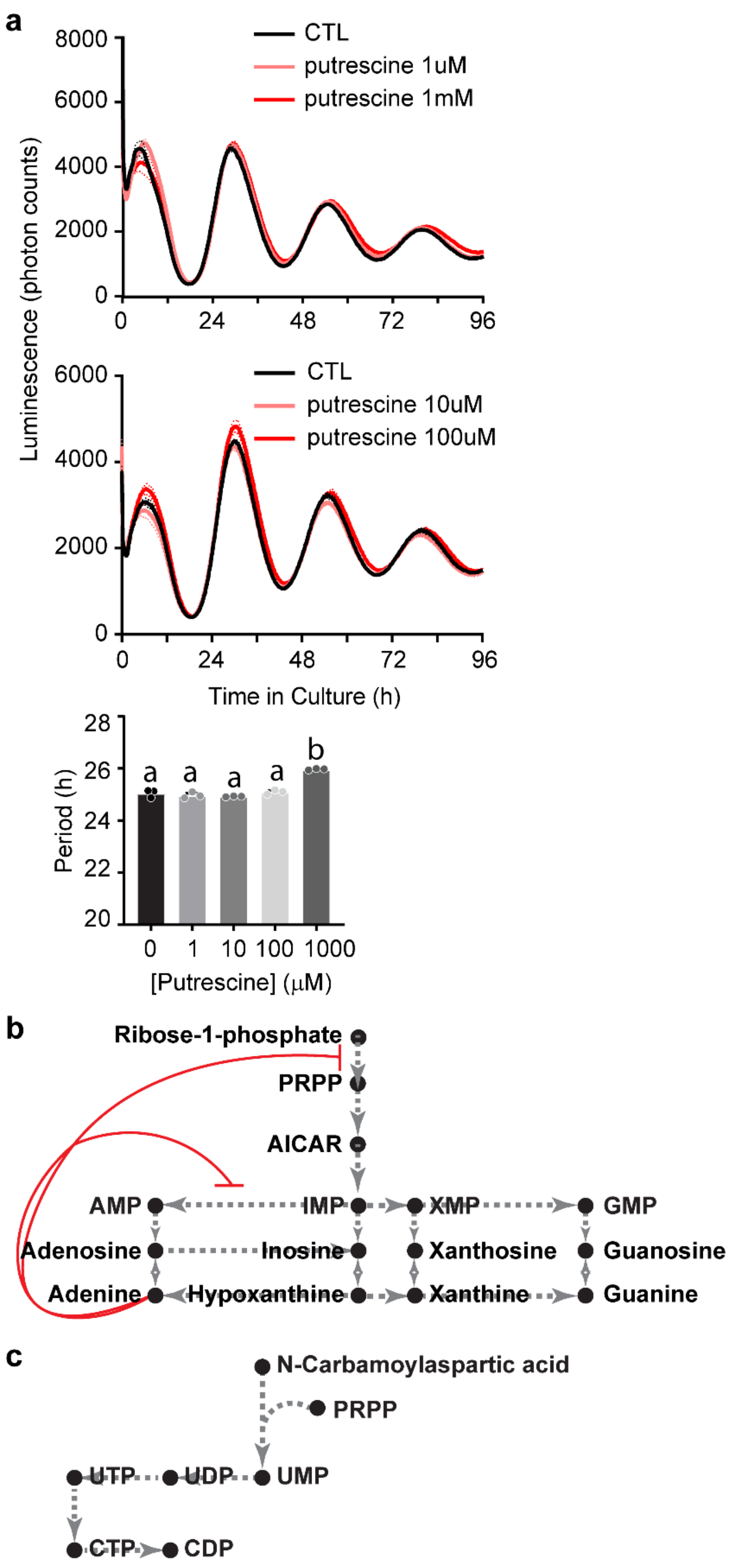

Supplementary Figure 2: Putrescine does not contribute to the effects of SAM on the circadian clock, but increased adenine explains changes in other nucleotides.

a, Putrescine only causes a mild period lengthening at 1 mM. Data shown are mean +/- SEM of 3 biological replicates (culture wells). Data shown as bar graphs analysed by One-Way ANOVA followed by Bonferroni multiple comparison test, a vs. b $p<0.0001$. **b**, Simplified purine nucleotides synthesis pathway, in which excess adenine inhibits the committing step of *de novo* purine synthesis

to phosphoribosyl pyrophosphate (PRPP) and its own branch of the pathway to AMP. **c**, Simplified pyrimidine nucleotides synthesis pathway, requiring PRPP.

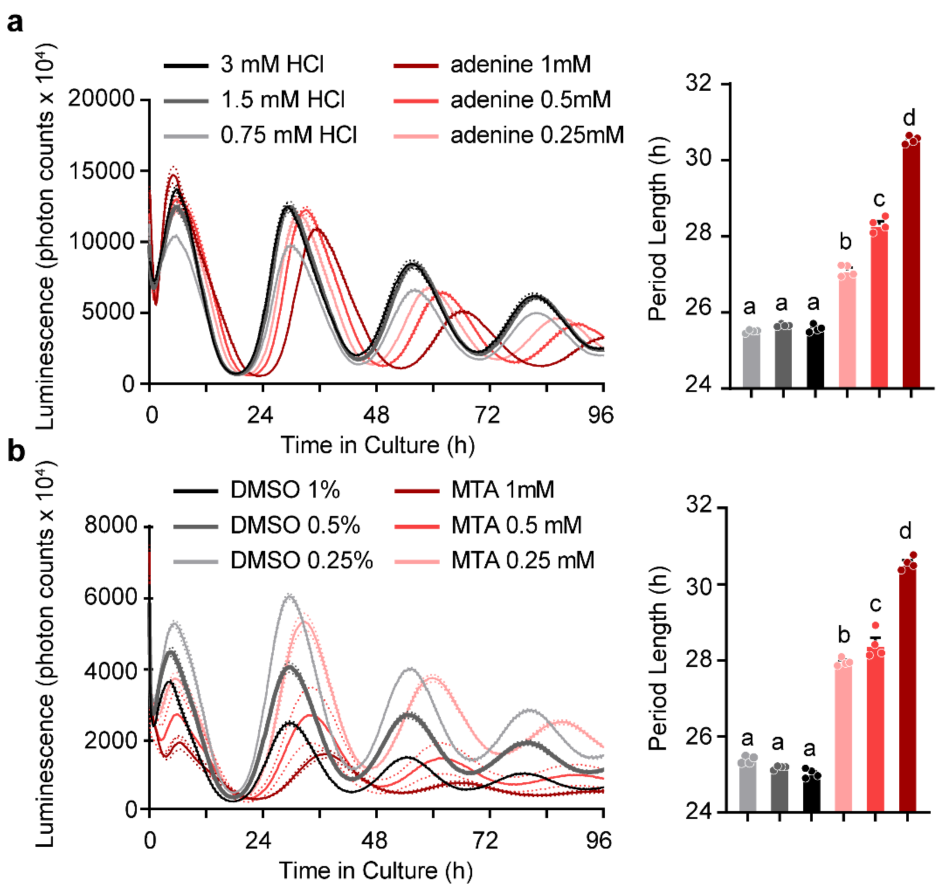

Supplementary Figure 3: MTA and adenine lengthen the circadian period.

a, PER2::LUC oscillations in mouse embryonic fibroblasts treated with increasing concentration of adenine or its vehicle HCl. Period length of the oscillations is shown on the right. **b**, PER2::LUC oscillations in mouse embryonic fibroblasts treated with increasing concentration of MTA or its vehicle DMSO. Period length of the oscillations is shown on the right. All data show mean +/- SEM of n = 4 cell culture wells. Data shown as bar graphs analyzed by One-Way ANOVA followed by Bonferroni multiple comparison test, a vs. b vs. c vs. d (labels at the top of each bar) at least $p<0.05$.

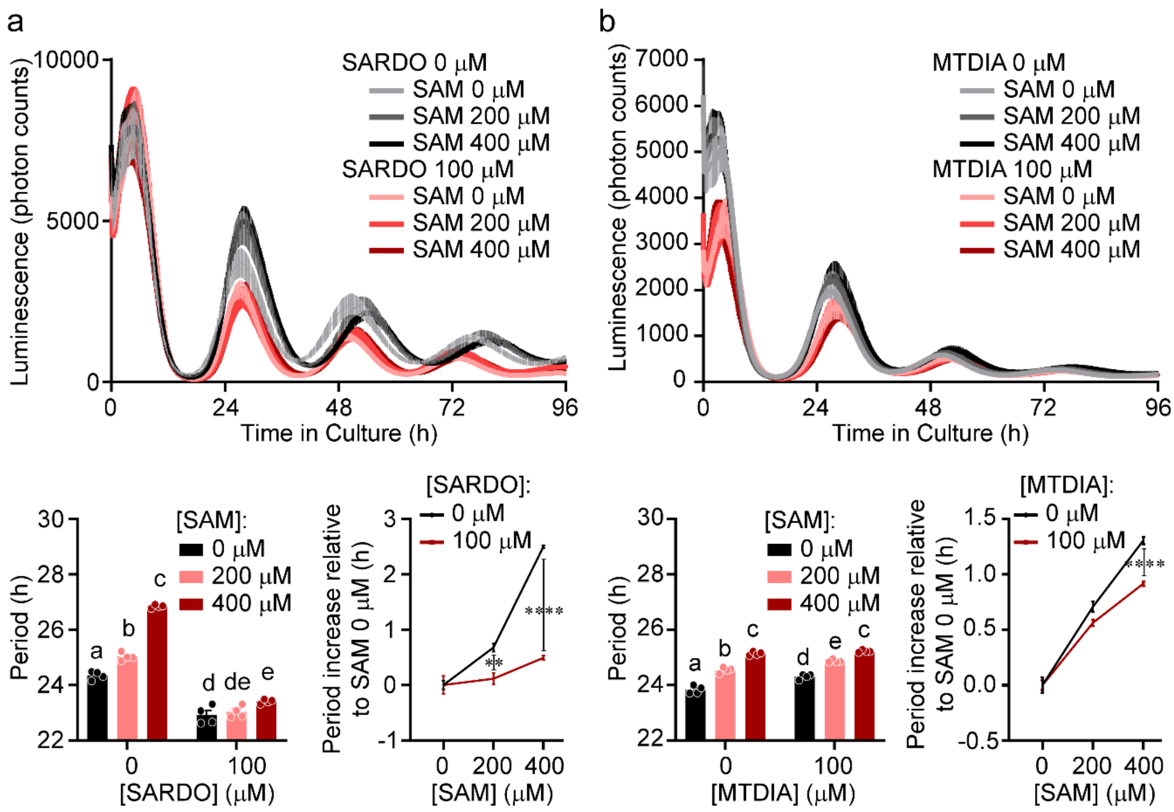

Supplementary Figure 4: Inhibition of the methionine salvage pathway rescues the cells from SAM. **a**, PER2::LUC oscillations in mouse embryonic fibroblasts treated with increasing SAM concentrations in the presence or absence of sardomozide (SARDO) or its vehicle (water). Period length of the oscillations is shown on the bottom left, and the increase in period on the bottom right. Period data analyzed by Two-Way ANOVA (effects of SAM, $p<0.0001$; of SARDO, $p<0.0001$; of interaction $p<0.0001$) followed by Bonferroni multiple comparison test, a vs. b vs. c vs. d vs. e (labels at the top of each bar) at least $p<0.05$. Period difference data analyzed by Two-Way ANOVA followed by Bonferroni multiple comparison test; **, $p=0.0014$; ****, $p<0.0001$. **b**, PER2::LUC oscillations in mouse embryonic fibroblasts treated with increasing SAM concentrations in the presence or absence of MTDIA or its vehicle (DMSO). Period length of the oscillations is shown on the bottom left, and the increase in period on the bottom right. Period data analyzed by Two-Way ANOVA (effect of SAM, $p<0.0001$; of MTDIA, $p=0.0001$; of interaction $p=0.0012$) followed by Bonferroni multiple comparison test, a vs. b vs. c vs. d vs. e (labels at the top of each bar) at least $p<0.05$. Period difference data analyzed by Two-Way ANOVA followed by Bonferroni multiple comparison test; ****, $p<0.0001$. All data show mean +/- SEM of n = 4 cell culture dishes.

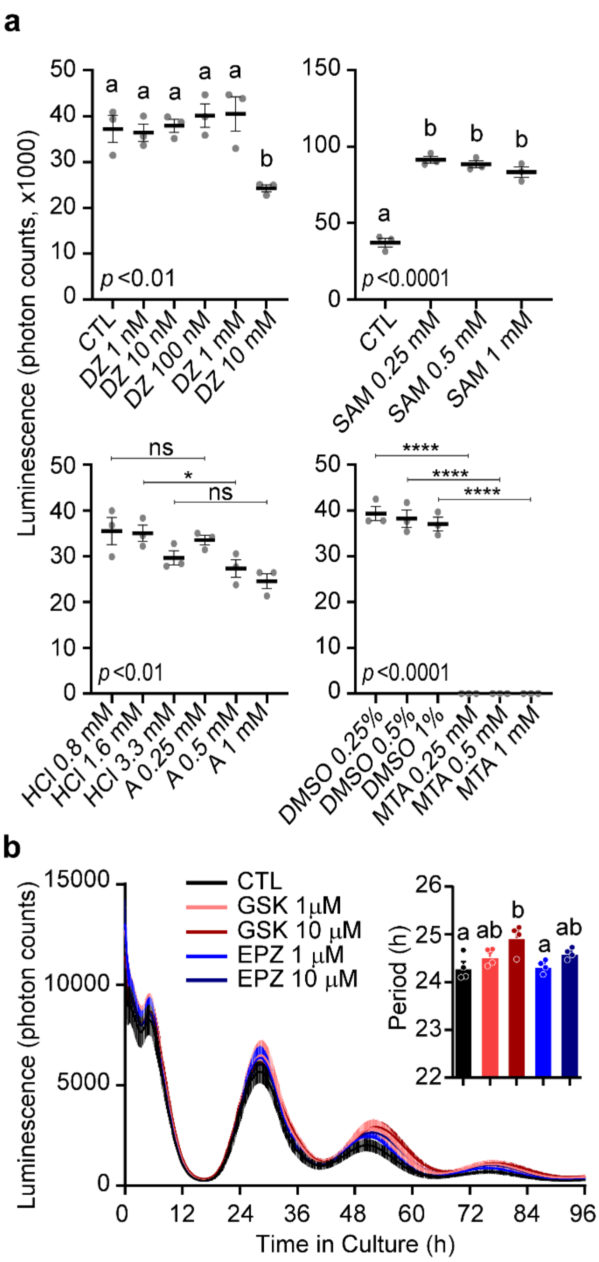

Supplementary Figure 5: Effects of direct inhibition of PRMT5 and adenosine + pentostatin on the circadian period.

a, *In vitro* PRMT5 enzymatic assays shows that MTA is a direct PRMT5 inhibitor. Adenine and DZ did not have clear effects. SAM in contrast stimulated PRMT5 activity on the H4R3 substrate. Data show mean luminescence +/- SEM of n = 4 immunoassay wells. HCl was used as a vehicle for adenine (A), DMSO for MTA, and water for DZ and SAM. DZ and SAM treatments analyzed by One-Way ANOVA followed by Bonferroni multiple comparison test, a vs. b (labels at the top of each bar) at least p<0.05; for adenine and MTA, only the indicated comparisons were made in Bonferroni multiple comparison test. **b**, Known PRMT5 specific inhibitors GSK591 and EPZ015666 only have mild effects on the circadian period in PER2::LUC mouse embryonic fibroblasts. Data show mean luminescence +/- SEM of n = 4 cell culture wells. Insert shows analysis of mean period +/- SEM by One-Way ANOVA followed by Bonferroni multiple comparison test, a vs. b (labels at the top of each bar) at least p<0.05.

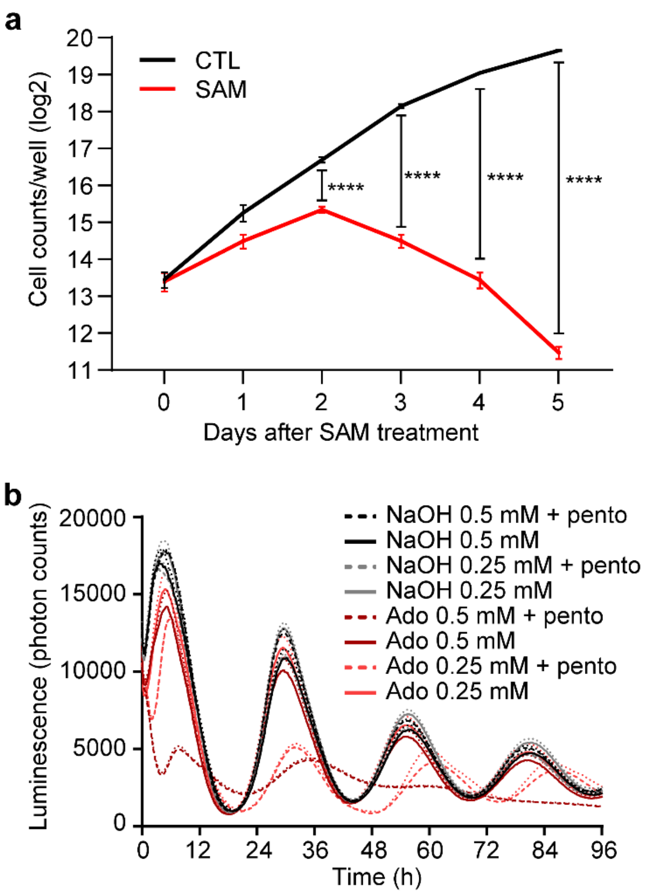

Supplementary Figure 6: Effects of SAM on cell proliferation and survival, and of adenosine on circadian rhythms when ADA is inhibited.

a, PER2::LUC mouse embryonic fibroblasts plated at low density were treated with 1 mM SAM or vehicle and counted every day for 5 days. Data show mean cell counts +/- SEM of n = 4 wells. Water was used as a vehicle for SAM. Treatment and cell growth analyzed by Two-Way ANOVA (all sources of variations p<0.0001) followed by Bonferroni multiple comparison test, **** p<0.0001. **b**, While adenosine (Ado) and 10 µM pentostatin (Nipent®) (pento) alone have no effect on the circadian period in PER2::LUC mouse embryonic fibroblasts (mean +/- SEM: Ado 0.25 mM, 25.26 +/-0.025h; Ado 0.5 mM, 25.21 +/-0.027h; NaOH 0.25 mM, 25.30 +/-0.052h; NaOH 0.5 mM, 25.16 +/-0.03h; NaOH 0.25 mM + pento, 25.14 +/-0.025h; NaOH 0.5 mM + pento, 25.14 +/-0.015h), together they dramatically and significantly lengthen the period (Ado 0.25 mM + pento, 28.83 +/- 1.29h; Ado 0.5 mM + pento, 28.81 +/-0.081h; all comparison between other groups p<0.01 by One-Way ANOVA followed by Bonferroni multiple comparison test). Period graph is not shown. Data show mean luminescence +/- SEM of n = 3 cell culture wells. NaOH was used as a vehicle for Ado.

Fig. 4e

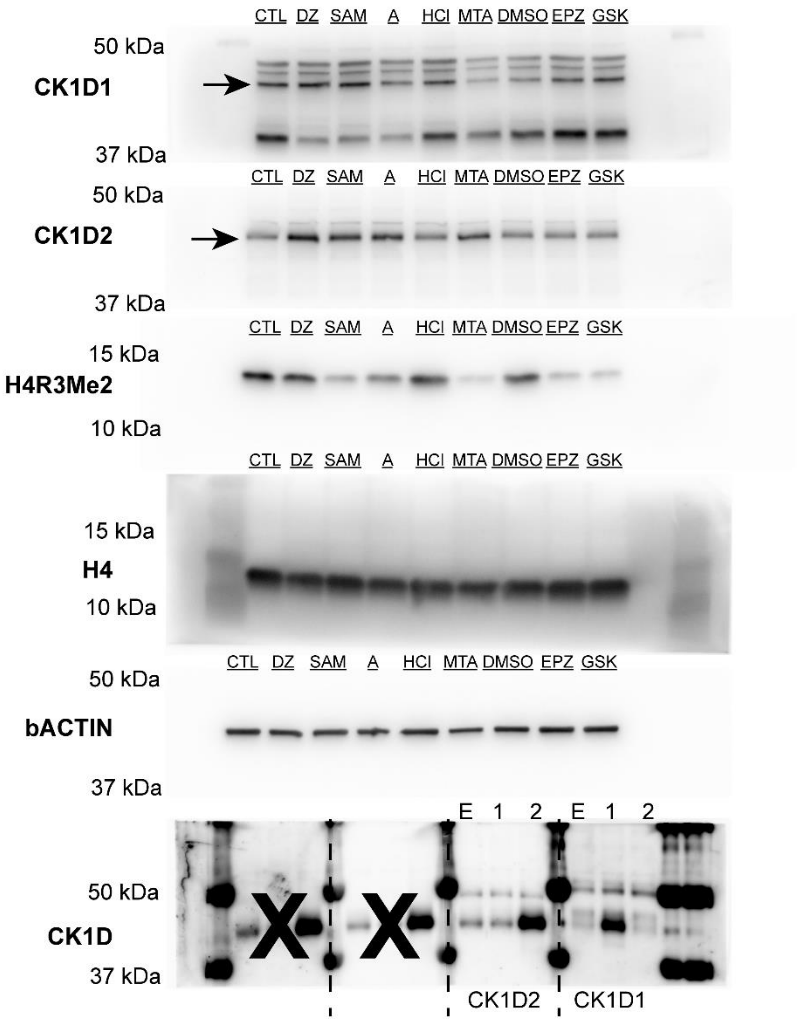

Fig. 4g

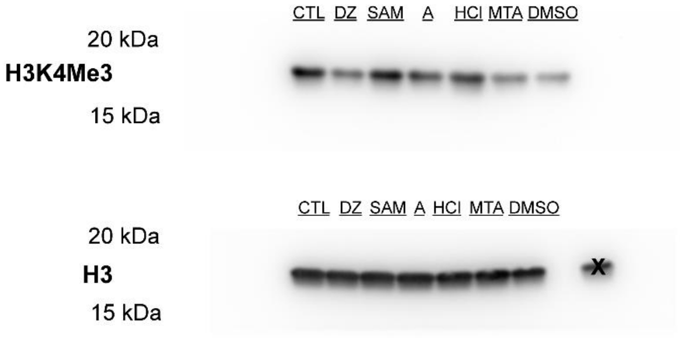

Fig. 4d

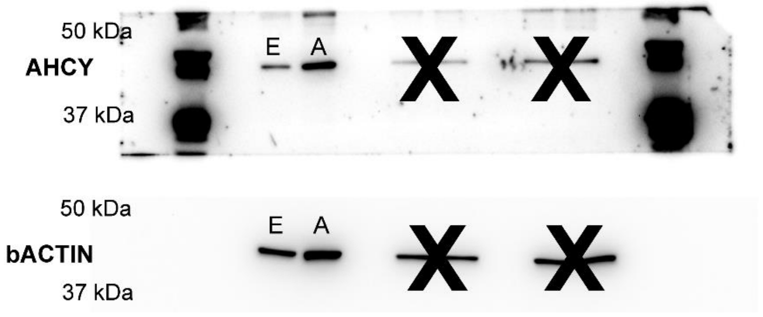

Supplementary Figure 7: Uncropped membranes.

For CK1D blot, the membrane was cut along the dotted lines and each section was incubated with the antibody indicated at the bottom. For all blots shown, bands/sections marked with a X are not relevant.

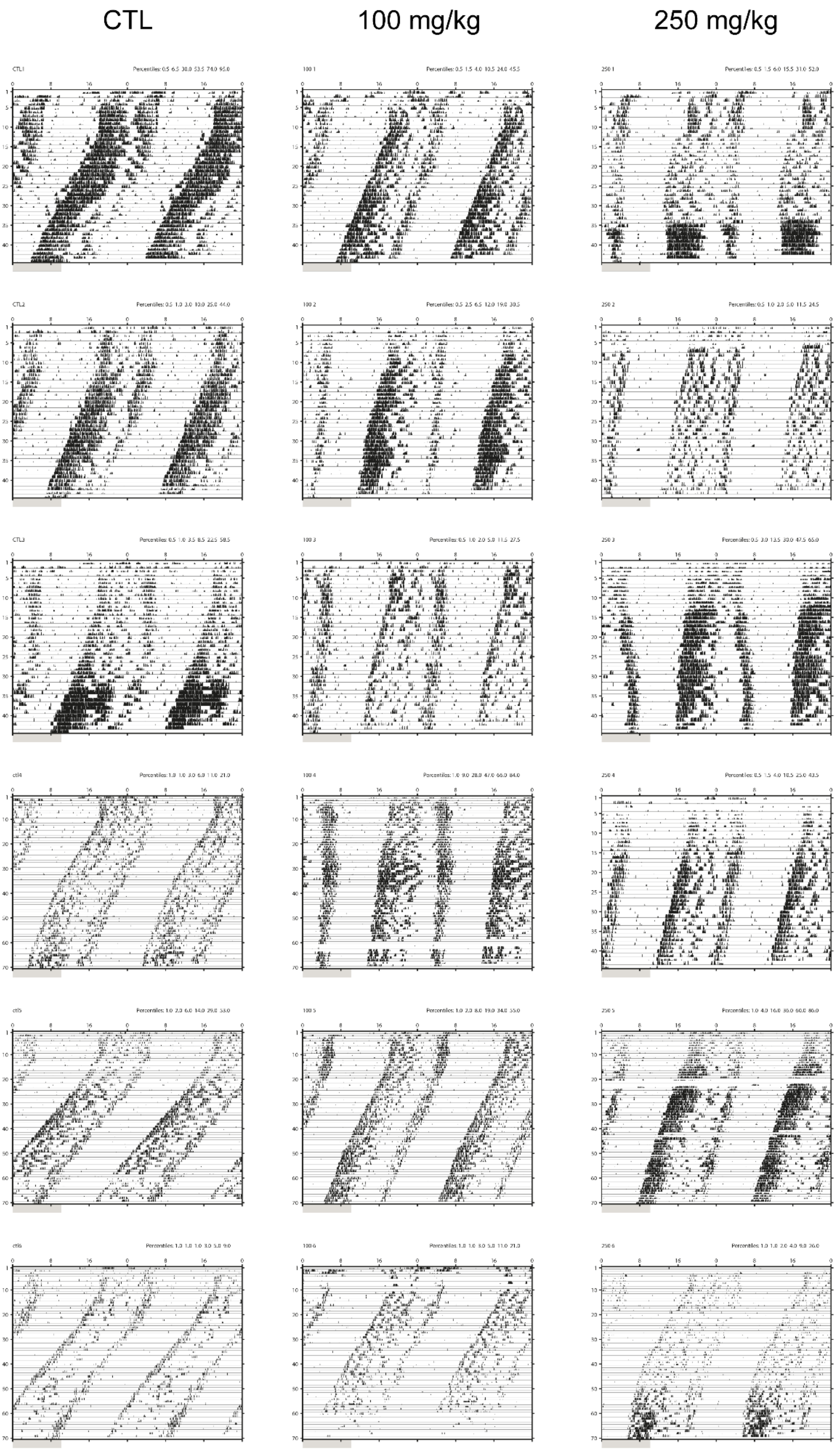

Supplementary Figure 8: Actograms

Raw actograms from all animals, treated as indicated above each column of actograms.
