# Supplementary material for: Excess S-adenosylmethionine inhibits methylation via catabolism to adenine
Source: Commun Biol. 2022 Apr 5;5:313. doi: 10.1038/s42003-022-03280-5 (PMC8983724; doi:10.1038/s42003-022-03280-5)
Supplement: Supplementary file 3 — Description of Additional Supplementary Files [file 42003_2022_3280_MOESM3_ESM.pdf]

Description of Additional Supplementary Files

File name: Supplementary Data 1
Description: raw metabolome data and analysis. Contains all metabolites included in the analysis. Only known and significant metabolites in the Metaboanalyst analysis were included in the heatmap shown in Figure2a.

File name: Supplementary Data 2
Description: raw metabolome data and analysis. Contains all metabolites included in the analysis. Only known and significant metabolites in the Metaboanalyst analysis were included in the heatmap shown in Figure 3a.

File name: Supplementary Data 3
Description: results from EdgeR analysis of RNAseq data shown in Figure 5b. One tab shows normalised expression values (logCPM); the other tabs correspond to each pairwise comparison against control group (CTL).

File name: Supplementary Data 4
Description: GOEnrichment Gene ontology analysis using genes significantly regulated against control group in each treatment.
