# Supplementary material for: Excess S-adenosylmethionine inhibits methylation via catabolism to adenine
Source: Commun Biol. 2022 Apr 5;5:313. doi: 10.1038/s42003-022-03280-5 (PMC8983724; doi:10.1038/s42003-022-03280-5)
Supplement: Supplementary file 8 — Reporting Summary [file 42003_2022_3280_MOESM8_ESM.pdf]

nature portfolio

Corresponding author(s): Jean-Michel Fustin

Last updated by author(s): Feb 3, 2022

Reporting Summary

Nature Portfolio wishes to improve the reproducibility of the work that we publish. This form provides structure for consistency and transparency in reporting. For further information on Nature Portfolio policies, see our Editorial Policies and the Editorial Policy Checklist.

Statistics

For all statistical analyses, confirm that the following items are present in the figure legend, table legend, main text, or Methods section.

n/a	Confirmed	
☐	☒	The exact sample size (*n*) for each experimental group/condition, given as a discrete number and unit of measurement
☐	☒	A statement on whether measurements were taken from distinct samples or whether the same sample was measured repeatedly
☐	☒	The statistical test(s) used AND whether they are one- or two-sided<br>*Only common tests should be described solely by name; describe more complex techniques in the Methods section.*
☒	☐	A description of all covariates tested
☒	☐	A description of any assumptions or corrections, such as tests of normality and adjustment for multiple comparisons
☐	☒	A full description of the statistical parameters including central tendency (e.g. means) or other basic estimates (e.g. regression coefficient) AND variation (e.g. standard deviation) or associated estimates of uncertainty (e.g. confidence intervals)
☒	☐	For null hypothesis testing, the test statistic (e.g. *F*, *t*, *r*) with confidence intervals, effect sizes, degrees of freedom and *P* value noted<br>*Give P values as exact values whenever suitable.*
☒	☐	For Bayesian analysis, information on the choice of priors and Markov chain Monte Carlo settings
☒	☐	For hierarchical and complex designs, identification of the appropriate level for tests and full reporting of outcomes
☒	☐	Estimates of effect sizes (e.g. Cohen's *d*, Pearson's *r*), indicating how they were calculated

Our web collection on statistics for biologists contains articles on many of the points above.

Software and code

Policy information about availability of computer code

Data collection

Actograms and period estimates (chi-square periodogram) were acquired with Clocklab (Actimetrics).
Proteins were detected using chemiluminescent substrate (Amersham ECL-Prime), pictures of membranes were acquired with a G:Box (Syngene).
Liquid chromatography-mass spectrometry analysis was performed using a Thermo-Fisher Ultimate 3000 HPLC system consisting of an HPG-3400RS high pressure gradient pump, TCC 3000SD column compartment and WPS 3000 Autosampler, coupled to a SCIEX 6600 TripleTOF Q-TOF mass spectrometer with TurboV ion source. The system was controlled by SCIEX Analyst 1.7.1, DCMS Link and Chromeleon Xpress software.
For metabolome analysis, peaks detected in CE-TOFMS analysis were extracted using automatic integration software (MasterHands ver.2.17.1.11 developed at Keio University)66 and those in CE-QqQMS analysis were extracted using automatic integration software (MassHunter Quantitative Analysis B.06.00 Agilent Technologies) in order to obtain peak information including m/z, migration time (MT), and peak area.

Data analysis

Integrated intensities from immunoblotting membranes were quantified with ImageJ available at https://imagej.nih.gov/.
For metabolite quantification, acquired data was processed in MultiQuant 3.0.2. Peaks from MS1 and MS2 data were picked and matched against a metabolite library of 235 standards, based on retention time and mass error of ± 0.025 Da. Data exported from MultiQuant 3.0.2 was further sorted, filtered and scored using a custom VBA macro in Excel, based on presence, peak area and coelution of precursor and fragment ions.
Metabolome data were analysed using Metaboanalyst 5.0 (https://www.metaboanalyst.ca/)
Raw reads from RNA-Seq data were uploaded to the Galaxy server70 and its instance at the University of Manchester (https://centaurus.itservices.manchester.ac.uk/), QCed and trimmed using Trimmomatic71, then aligned to the mouse genome GRCm39 guided by the vM27.annotations from GENCODE72 using HISAT2 aligner73. Transcripts were assembled and quantified using Stringtie74,75, and differential expression analysis was performed using edgeR on genes with a CPM of at least 1 in all samples76,77. Hierarchical clustering and heatmap were performed on GenePettern78. GOEnrichment (https://github.com/DanFaria/GOEnrichment) was performed on the Galaxy

server.

For manuscripts utilizing custom algorithms or software that are central to the research but not yet described in published literature, software must be made available to editors and reviewers. We strongly encourage code deposition in a community repository (e.g. GitHub). See the Nature Portfolio guidelines for submitting code & software for further information.

Data

Policy information about availability of data

All manuscripts must include a data availability statement. This statement should provide the following information, where applicable:
- Accession codes, unique identifiers, or web links for publicly available datasets
- A description of any restrictions on data availability
- For clinical datasets or third party data, please ensure that the statement adheres to our policy

RNAseq data and associated files have been deposited in NCBI's Gene Expression Omnibus repository, accession number GSE184525. Uncropped blot membranes and actograms for all animals are provided as Source data.

Field-specific reporting

Please select the one below that is the best fit for your research. If you are not sure, read the appropriate sections before making your selection.

☒ Life sciences ☐ Behavioural & social sciences ☐ Ecological, evolutionary & environmental sciences

For a reference copy of the document with all sections, see nature.com/documents/nr-reporting-summary-flat.pdf

Life sciences study design

All studies must disclose on these points even when the disclosure is negative.

Sample size	For all experiments sample size was selected considering the inherent variability of each method used to collect the data, from our experience with such methods.
Data exclusions	No data were excluded.
Replication	All results shown are either representative of at least 3 replicate experiments (in vitro) or the accumulation of results from 3 independent experiments (in vivo).
Randomization	Typically, treatment of cells was randomly allocated to wells of a cell culture plate or to dishes in an incubator, and mice were randomly allocated to cages, diet and position within light-tight cabinets.
Blinding	Blinding is not relevant to this study because all methods of quantification used are not influenced by the researcher's subjectivity, i.e. Chi square periodogram.

Reporting for specific materials, systems and methods

We require information from authors about some types of materials, experimental systems and methods used in many studies. Here, indicate whether each material, system or method listed is relevant to your study. If you are not sure if a list item applies to your research, read the appropriate section before selecting a response.

Materials & experimental systems

n/a	Involved in the study	
☐	☒	Antibodies
☐	☒	Eukaryotic cell lines
☒	☐	Palaeontology and archaeology
☐	☒	Animals and other organisms
☒	☐	Human research participants
☒	☐	Clinical data
☒	☐	Dual use research of concern

Methods

n/a	Involved in the study	
☒	☐	ChIP-seq
☒	☐	Flow cytometry
☒	☐	MRI-based neuroimaging

Antibodies

Antibodies used	AHCY, ProteinTech 10757-2-AP, 1:750; H4R3Me2, ABCAM ab5823, 1:1000; H4, ABCAM ab10158, 1:1000 and Cell Signalling 13919S, 1:1000; Actin, Sigma A5441, 1:5000; CK1D1 and CK1D2, Yanaihara Research Institute, 1:1000; Anti-rabbit HRP secondary antibodies, Amersham NA934, 1:10,000 and anti-mouse, Amersham NA931, 1:50,000. Multiple lots of commercially available antibodies were used in these studies.

Validation	All commercially available antibodies were selected according to the number of reliable publications (our own, from our collaborators or from known scientists) reporting their use, and further tested using positive (transfection experiments) and/or negative (knock-out or knock-in) controls.<br>The specificity of our anti-CK1D1 and anti-CK1D2 antibodies, produced by the Yanaihara Research Institute in Japan, was ascertained using respective positive controls (CK1D1 or CK1D2 over-expression).

Eukaryotic cell lines

Policy information about cell lines

Cell line source(s)	PER2::LUC heterozygous knock-in mice.
Authentication	Cell lines were prepared by ourselves, well characterised, and did not require authentication.
Mycoplasma contamination	Lack of mycoplasma contamination checked before experiments using LOOKOUT MYCOPLASMA PCR DETECTION KIT, Merck.
Commonly misidentified lines (See ICLAC register)	We did not use commonly misidentified cell lines.

Animals and other organisms

Policy information about studies involving animals; ARRIVE guidelines recommended for reporting animal research

Laboratory animals	Mouse, C57BL/6J from Charles River (UK).
Wild animals	The study did not use wild animals.
Field-collected samples	The study did not involve field-collected samples.
Ethics oversight	Animal experiments were licensed under the Animals (Scientific Procedures) Act of 1986 (UK) and were approved by the animal welfare committees at the University of Manchester.

Note that full information on the approval of the study protocol must also be provided in the manuscript.
